# The Hub-and-Spoke Management of Glaucoma

**DOI:** 10.3389/fnins.2020.00180

**Published:** 2020-03-17

**Authors:** Raffaele Nuzzi, Paola Marolo, Alessia Nuzzi

**Affiliations:** ^1^Eye Clinic Section, Department of Surgical Sciences, University of Turin, Turin, Italy; ^2^Department of Clinical Sciences and Community Health, Eye Clinic San Giuseppe Hospital, IRCCS Multimedica, University of Milan, Milan, Italy

**Keywords:** glaucoma, prevention, screening, diagnosis, hub-and-spoke, management, network

## Abstract

Glaucoma is an extremely significant public health issue, since it is the most common cause of irreversible blindness worldwide, nevertheless it is still widely undiagnosed because of its devious nature. Glaucoma diagnosis criteria are well-defined and have to be strictly observed and recognized: the earlier the disease is diagnosed, the earlier the patient can undergo the most suitable treatment, the better can be the prognosis. The three levels of prevention are essential in the approach to the disease and its pathophysiological features make it eligible for screening. This review provides an overview of the current state of the art in glaucoma management, starting from its prevention and coming to the hub-and-spoke organization. This model applied to glaucoma aims to direct patients toward professional and not professional figures who may guide them in integrated care pathway. This path should be designed in accordance with best practice to coordinate glaucoma prevention, diagnosis, treatment and follow up with the best cost-benefit ratio, protecting both the interests of the patient and of the society.

## Introduction

*Glaucoma* is a very ancient term and, as the etymology indicates, was used to denote the bluish-green color of pupil, that is typical of the end stage of this disease. Until the 18th century, glaucoma was mistaken with cataract: Brisseau was the first in 1700 to prove that glaucoma and cataract differ greatly from one to another (Boles Carenini, 1990).

As it is widely known, glaucoma is the most common cause of irreversible blindness and the second cause of visual impairment after cataract worldwide (Resnikoff et al., 2004; Quigley and Broman, 2006; Bourne et al., 2013). Nevertheless, over one-third of cases remain undiagnosed (Whitson, 2007).

The estimated prevalence of the disease is 2.5% in Caucasian population over 40 years of age (Bonomi et al., 1998). It was predicted that glaucoma would have affected 60 million patients worldwide and 8.4 million of these would have been blind in 2010, while there will be 79.6 million patients affected and 11.1 million blind in 2020 (Quigley and Broman, 2006). Moreover, it is calculated that glaucoma will affect 111.8 million people in 2040 worldwide (Tham et al., 2014) and, according to the National Eye Institute, the number is set to increase in 2050 (Wojcik-Gryciuk et al., 2015). But what is meant by glaucoma? Glaucoma is a progressive optic neuropathy characterized by peculiar morphological abnormalities of the optic nerve head (ONH) and retinal nerve fiber layer (RNFL) in absence of other ocular pathologies (Guidelines, 2017). The progressive loss of retinal ganglion cells (RGC) leads to an increasing and irreversible visual field defects, outlining the peripherical area and then the central fixation points in end-stages (Nuzzi and Tridico, 2017). This disease shows no early symptoms in most cases and patients are unaware (Quigley, 2011); if symptoms appear they are vague and can include headaches, severe eye pain, vomiting, hazy or blurred vision and rainbow-colored circles around bright lights. The overlap of several neuro-ophthalmologic conditions complicates further the diagnosis. Visual field defects, neuropsychiatric pathologies signs and not-progressive disorders can mimic glaucoma: in some of these cases, in addition to considering the intervention of a multidisciplinary team, functional and nuclear magnetic resonance imaging can be diriment and determinant (Balendra et al., 2015) for differential diagnosis and also to collect “marker images” of glaucomatous retinal fundus.

## Glaucoma Diagnosis

The risk factors of glaucoma are numerous: age, ethnicity, intraocular pressure (IOP), pseudoexfoliation syndrome (PEX), high myopia (greater than −3 diopters), thinner central corneal thickness (CCT), family history of glaucoma, low ocular perfusion pressure, drugs (steroids, antidepressants, calcium antagonists) (Giangiacomo et al., 2009) and there are many variables concerning diagnosis and evaluation of glaucoma progression.

**FIGURE 1:**
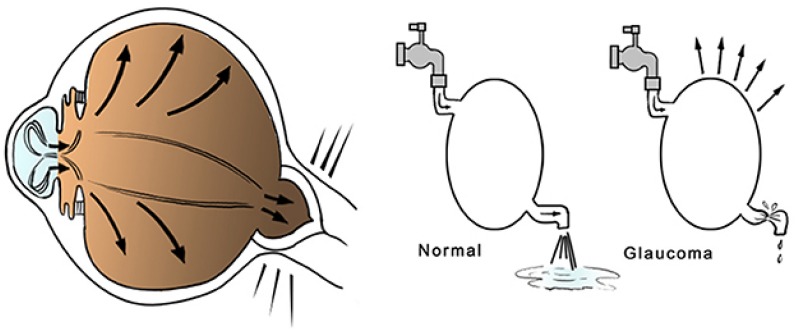
Diagram of glaucoma pathogenesis: the reduced outflow or the excessive production of aqueous humor is frequently associated with IOP elevation, which may generate pathological changes in the optic nerve fibers.

**FIGURE 2:**
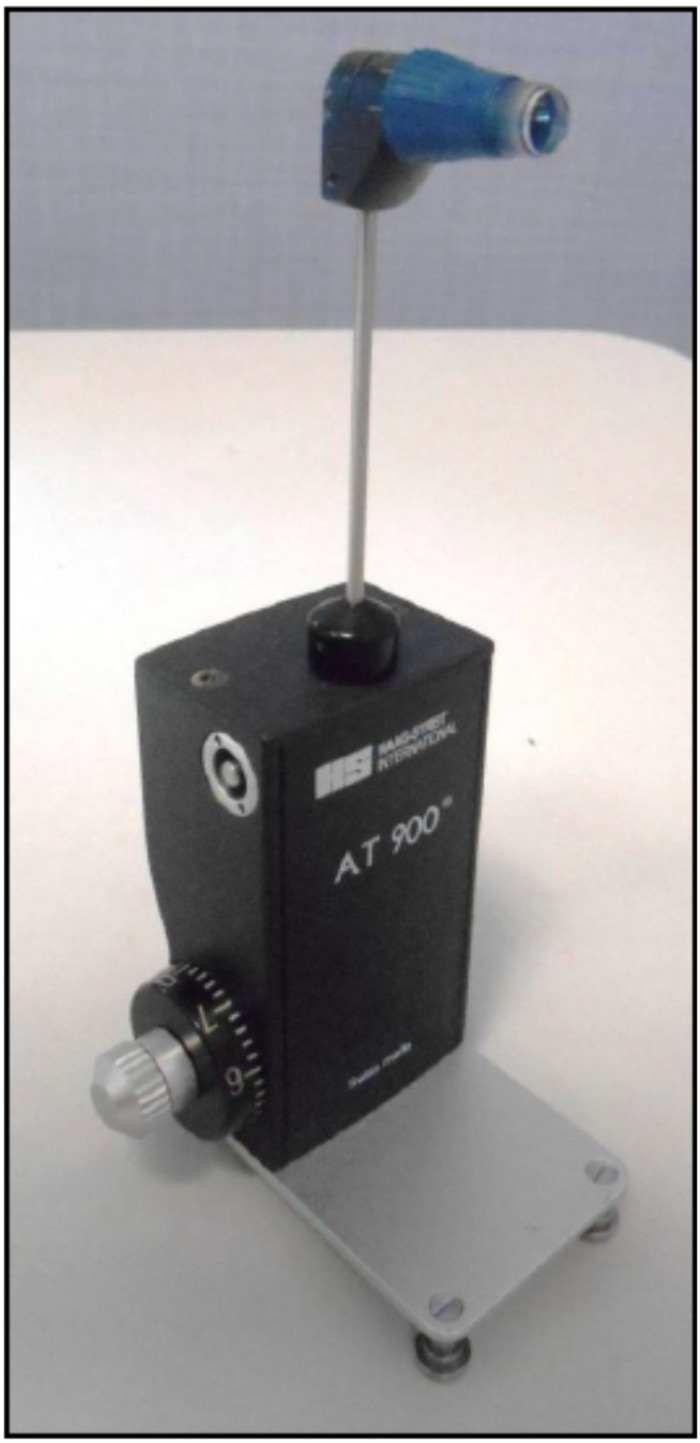
The Goldmann applanation tonometer: it shall be positioned on the slit lamp.

**FIGURE 3:**
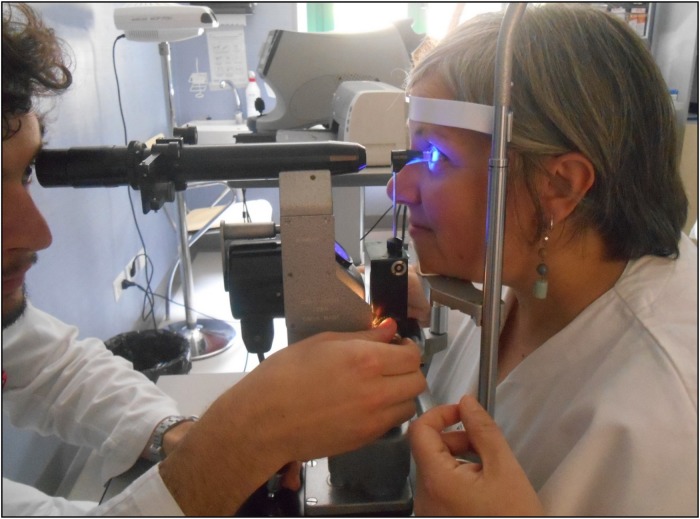
IOP measurement using the Goldmann tonometer. Written informed consent was obtained from the individuals for the publication of this image.

**FIGURE 4:**
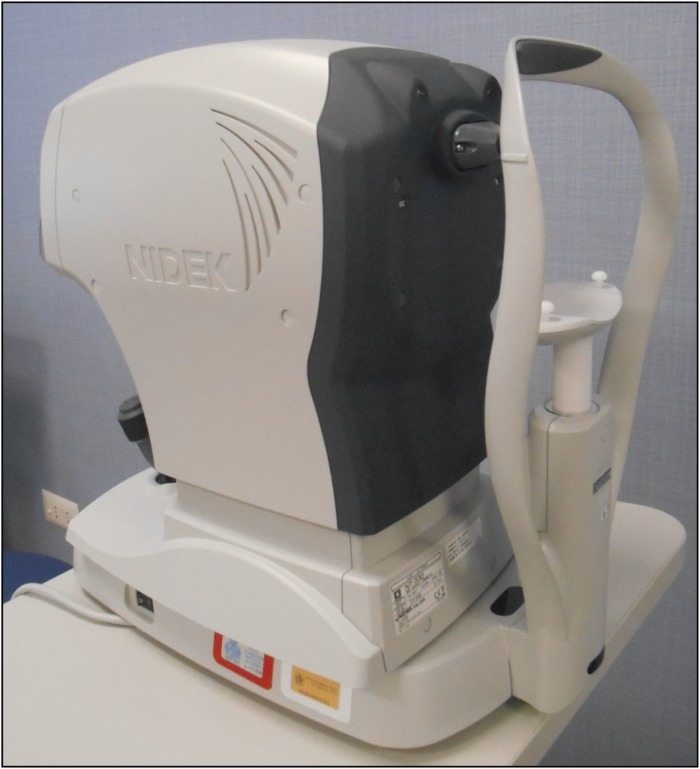
The non-contact air-puff tonometer.

The IOP is the main risk factor for the development of glaucoma and its progression and it is measured by tonometry (Figure 1). The mean IOP between adults is 15–16 mmHg with a standard deviation of 3.0 mmHg, but the presence of a high IOP (ocular hypertension) in absence of optic nerve or perimetry alterations does not necessarily mean glaucoma. However, it is estimated that about 10% of patients with ocular hypertension will develop glaucoma in 5 years (Burr et al., 2007). The IOP measurement can be repeated several times to create a daily tonometric curve in order to obtain a greater reliability (Mansouri and Weinreb, 2015). There are two main types of tonometers: contact and non-contact. The current reference standard is the Goldmann applanation tonometer (GAT) (Figures 2, 3), while alternative tonometers are: the non-contact air-puff tonometer (Figure 4), pneumatonometry, dynamic contour tonometry, ocular response analyzer, the Ocuton S tonometer, rebound tonometry (Icare) and Tono-Pen. The last two are portable and hand-held and, as the area of the contact with the cornea is small, can be used for patients with corneal diseases and surface irregularity. The non-contact air-puff tonometer gives a variable number of false positive even though does not require contact with apex of the cornea and anesthesia. For these characteristics, the non-contact air-puff tonometer is useful in mass screening; in case of doubt, it shall be supplemented with the Goldmann tonometer and the tonometric pen (Kouchaki et al., 2016).

**FIGURE 5:**
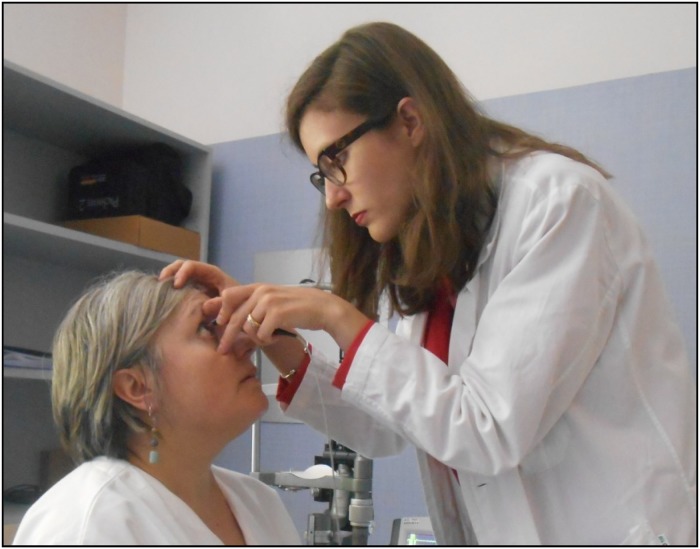
The execution of pachymetry: the ultrasound probe shall be placed on the corneal apex. Written informed consent was obtained from the individuals for the publication of this image.

**FIGURE 6:**
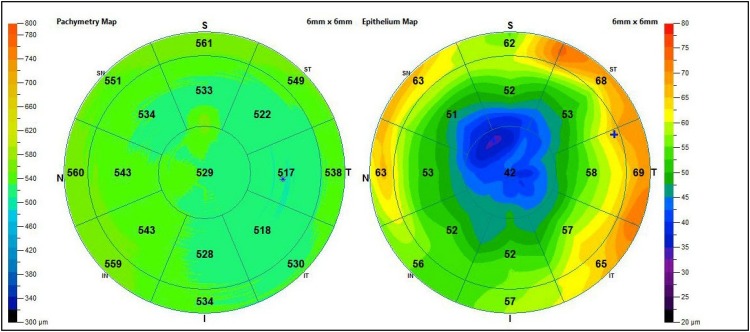
Pachymetry report: this has been obtained by an OCT execution of the anterior segment.

Corneal pachymetry is another significant test to perform at least once in life in the screening of all patients with risk factors or suspected glaucoma (Figure 5), because IOP value is directly related to corneal thickness. In fact, important correlations between corneal pachymetry and glaucoma have emerged unequivocally, due to the diffusion of refractive surgery and the related pre-op tests. Considering that the CCT in normal conditions is just over half a millimeter (540 ± micron), patients with thin cornea have a higher risk of development and progression of glaucomatous damage, while those with thick cornea are more protected from glaucomatous risk (Figure 6) (Gaspar et al., 2017; Belovay and Goldberg, 2018). However, it is important to remember that measurement of IOP by standard techniques is lower than the real value in patients with thin cornea and higher in those with thick cornea. In light of this, IOP can be underestimated or ignored in thin corneas, while it is much less alarming in thick corneas. Many corrective formula have been applied to calculate the real IOP, but there is no evidence to support a validated correction algorithm for GAT and CCT. Another aspect to notice, as mentioned above, is that ocular hypertension and glaucoma do not match; indeed, there is also a type of glaucoma “at low pressure” (especially in elderly population), where optic nerve alterations occur in the absence of high IOP. In this specific type, the presence of migraine, papillary hemorrhages, vascular alterations (in particular in the cephalic district) and nocturnal apneas should always be investigated (Mi et al., 2014; Killer and Pircher, 2018).

For differential diagnosis among the different types of glaucoma (open or closed angle), gonioscopy with or without indentation with direct or indirect viewing of the iridocorneal angle should be performed. The Spaeth gonioscopic grading system is the most detailed, but also Shaffer and Kanski grading systems are used, based on angle width and visibility of the structures. Predicting factors in detecting angular variations are high hypermetropia for closed-angle glaucoma, medium-high myopia and diabetes in the open-angle glaucoma cases.

Ophthalmoscopy, performed with an indirect non-contact fundus lens with sufficient magnification, allows to determine qualitative and quantitative evaluation of the optic nerve head and the RNFL. The examination should assess the shape of the neuroretinal rim, the appearance of the RNFL (best valued with a red-free photography), the possible presence of optic disc hemorrhages, the position of the vessels at the optic disc as well as parapapillary atrophy (alpha and beta zones). The cup/disc ratio (CDR) of the optic nerve head was used to determine glaucoma damage, but it widely depends on the size of the disc. Considering that individual variations are frequent, comparison with the contralateral optical papilla is essential: collecting photographic documentation of the appearance of each glaucomatous or suspected papilla and its evolution is helpful, in order to evaluate the stability or progression of the damage and the effectiveness of possible antiglaucomatous therapy. Furthermore, the morphology of the optic nerve head has an extremely high inter-individual variability: the clinicofunctional correlation is not always the same in different patients, especially in those with high-grade myopia.

Imaging instruments help to quantify the optic nerve damage and the glaucoma progression, using software based on normative databases: Heidelberg Retina Tomography (HRT), scanning laser polarimetry (GDx-ECC), optical coherence tomography (OCT) with study of ONH, RNFL, RGC and morphometric study of the angle.

**FIGURE 7:**
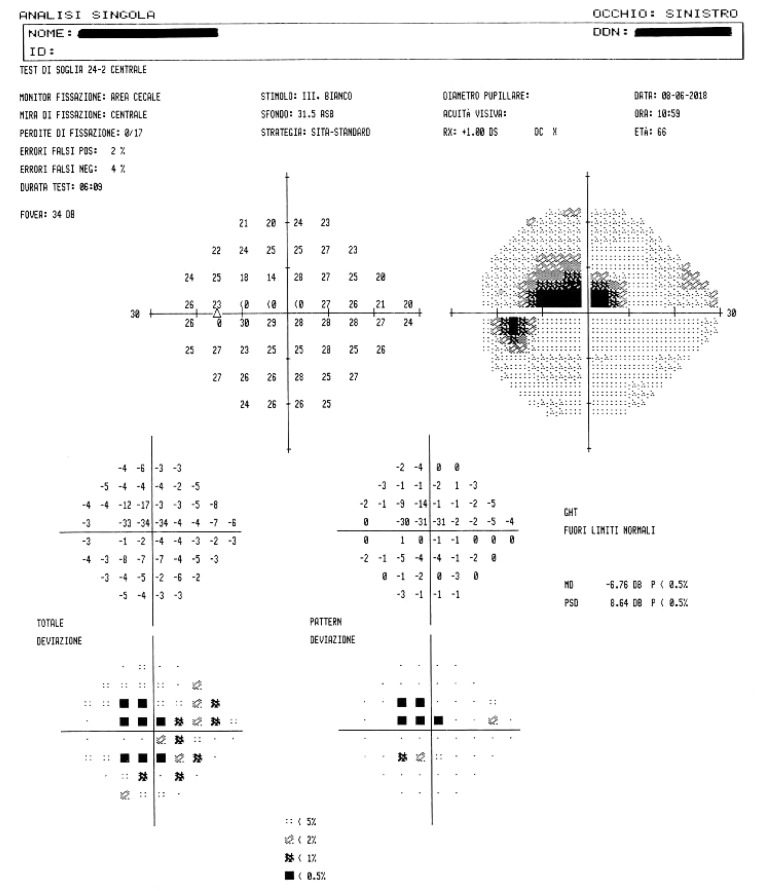
A visual field examination showing campimetric defects.

The gold standard of functional damage assessment is static computerized perimetry: this consists in a subjective examination, relatively long, which needs confirmation if it results altered (up to 80% of changes in the first visual field are not confirmed in a second time). This examination detects even minimal changes in visual field, it allows an accurate control of patient’s fixation and also a comparison of each examination with the previous ones of the same patient (Figure 7). The manual kinetic perimetry with Goldmann perimeter is still valid and may help in patients who are unable to perform automated perimetry, but it is an ineffective examination in exploring the paracentral area (the 30 central degrees), frequently affected by glaucoma, especially in the early stages of disease (Bowling and Kanski, 2016).

## Eye Diseases Prevention

Eye diseases lead to impaired quality of life, affecting all of its aspects and therefore have a serious impact on the health, as well as social and economic welfare of the individual and the society.

In order to find satisfactory solutions to these issues, “VISION 2020: The Right to Sight” has been the outcome of collaboration between the World Health Organization (WHO) and the International Agency for the Prevention of Blindness (IAPB) in 1999. It consists of the global proposal to fight and eliminate avoidable blindness by 2020. Nowadays, this project has been replaced by the Global Action Plan for the Prevention of Avoidable Blindness and Visual Impairment 2014–2019; however, its eye-opening message to promote “A world in which nobody is needlessly visually impaired, where those with unavoidable vision loss can achieve their full potential” has focused global attention on this humanitarian emergency and remains an appealing challenge (IAPB, 2020). Worldwide, 2.2 billion people have a vision impairment or blindness, half of whom have a preventable vision impairment (Flaxman et al., 2017; World Health Organization [WHO], 2019).

Accordingly, the diagnosis and early treatment of these pathologies would undoubtedly entail enormous benefits (Hubley and Gilbert, 2006; Bourne et al., 2017). In this perspective, the American Academy of Ophthalmology recommendations provide that anyone should undergo an eye examination at the age of 40:

1. one eye examination per year for patients over 65 years of age, with or without risk factors;
2. one eye examination every 2–4 years for patients aged 40 to 64, with or without risk factors;
3. one eye examination every 10 years for patients between 30 and 39 years of age, without risk factors, or one every 2–4 years if with risk factors; and
4. one eye examination every 10 years for patients between 20 and 29 years of age, without risk factors, or one every 3–5 years if with risk factors (Feder et al., 2016).

In this context, unequal literacy and access to facilities always have to be considered.

According to IAPB Onlus data, in the world these recommendations are not implemented and the collective sensitivity to eye health is poor: for example, 18% of Italian adults have never been visited by an ophthalmologist, 61% were visited once, but not checked in the last 5 years, 21% have had an eye examination in the last 5 years (Bausch+Lomb, 2012; IAPB, 2012).

Faced with these facts, in order to raise a greater responsiveness to these pathologies, all the institutions have to improve information and support prevention programs, for increasing the propensity of population to adhere to monitoring projects over time, which have to be invested in. As for any kind of medical condition, eye diseases prevention is divided into three levels.

Primary prevention aims at the healthy subject and consists in information and dissemination of basic notions about the disease. Its purpose is to prevent the disease from arising when it has not yet occurred and to act against environment and man (both individually and collectively). Some instruments of primary prevention in Italy promoted by IAPB ONLUS are the following: eye consultation toll-free number, The World Day of Vision, “The Ophthalmologist Says” forum, the “Social Ophthalmology” publication, the IAPB website (www.iapb.it). The Italian Ophthalmology Society (SOI) is also active in this field: the “For the citizen” website section, the “Together for the sight” foundation, the “Save the Vision” campaign. In glaucoma, primary prevention consists in making the disease known, as well as its risk factors. For example, subjects with narrow angle and high hypermetropia have an increased risk of developing acute angle-closure glaucoma, therefore it should be assessed whether adequate preventive monitoring of the IOP is necessary and possibly also the execution of a laser prophylactic iridotomy case by case basis (Yassur et al., 1979; He et al., 2019). Conversely, secondary prevention is directed to unaware affected people. Therefore, it corresponds to screening programs and it aims at early diagnosis to detect the disease when it is still asymptomatic; it can be done at individual or mass level. In glaucoma, secondary prevention shall be carried out mainly between the ages of 40 and 60.

Tertiary prevention is about aware affected people. It is intended to heal the underlying disease (with medical and/or surgical therapy), to manage its complications and to prevent progression.

Tertiary prevention is also the management of disability through pensions, benefits and allowances. Various approaches of rehabilitative therapy are an additional resource to treat the advanced stage of glaucoma, in particular educating how to use residual vision and training patients through visual stimulation strategies. Before scheduling these, the application of functional magnetic resonance imaging (fMRI) has proved to be particularly effective in evaluating plasticity of visual nervous system beforehand and for predictive purpose (Mastropasqua et al., 2015). Regarding the current research, promising related aspects concern cellular biological rehabilitation or implantations, which could be epiretinal, intraretinal, into the iridocorneal angle (sclero-corneal trabeculate) and at the level of the intraorbital optic nerve (also simultaneously).

In the panorama of glaucoma prevention, the congenital glaucoma deserves a separate discussion, because its pathophysiological mechanisms are still unclear and so early diagnosis (secondary prevention) represents the first bulwark against the disease. Primary congenital glaucoma (PCG) represents the majority of the pediatric glaucoma cases and it is due to incomplete development of trabecular meshwork before and/or after birth. Most cases are bilateral and diagnosed within the first year of life (>75%), more frequent in males and related to family history. It is rare and can be easily misdiagnosed and undetected. The earlier it is diagnosed, the earlier the patient can undergo angle surgery, the better can be the prognosis. The prognosis is better if diagnosed between 3 and 12 months of age (Papadopoulos and Khaw, 2007; Winreb and Papadopoulos, 2013). Care must be taken to epiphora, photophobia and blepharospasm in children below 1 year. In this affection eyes are larger than normal and severe cases show buphthalmos (Walton and Katsavounidou, 2005). In relation to juvenile open angle glaucoma (JOAG), children with family history should undergo more frequent ophthalmologic controls. A pediatric glaucoma patient has to be evaluated with appropriate instruments and examination under general anesthesia can be useful. Children older than 6 months are more easily evaluated under general anesthesia, but IOP can be falsely lowered. Finally, patients with pediatric glaucoma require lifelong follow-up (de Silva et al., 2011).

## Glaucoma Screening

Glaucoma affects the health-related quality of life in many ways: risk of unemployment, social issues with negative impact on relationships, diminished ability to read and drive, loss of independence in activities as sports and hobbies are typically associated with the progression of the disease. Furthermore, visual field loss and blindness associated with glaucoma have a significant economic impact on society (Nuzzi and Tridico, 2017), particularly on healthcare. In the United States, it was calculated that health expenditure for glaucoma amounts to more than $1.5 billion annually (Wojcik-Gryciuk et al., 2015). In Europe, there were an estimated 9.25 million people affected by glaucoma in 2001 (Michelson and Groh, 2001). In accordance with United States, direct medical costs for this disease is also high in European countries, because they can oscillate between € 455 and € 969 per person a year, not counting indirect costs related to low vision care and vision rehabilitation therapies (Traverso et al., 2005). Moreover, the world’s population is aging and this factor increase the frequency of chronic diseases as hypertension and diabetes, which have been demonstrated to contribute to glaucoma and high healthcare costs (Zhao et al., 2015; Song et al., 2016).

Therefore, glaucoma is a real issue of public health. In Italy, its inclusion in Essential Levels of Assistance (LEA) “has been defined fundamental” as mentioned in the Ministerial Decree DPCM n. 26/2001. Primary open-angle glaucoma (POAG) has the ideal characteristics for early diagnosis: it is a chronic disease with high prevalence, it is asymptomatic in the early stages and has a long latency period. The natural history of the disease is known, its diagnosis is easy and non-invasive, its treatment is possible and its prognosis is better as soon as it is diagnosed. Three are the main approaches to screening patients for POAG: IOP measurement, ONH and RNFL assessment and evaluating visual field, either alone or in combination. Despite these characteristics, however, there is no active screening program in Italy at national level, as well as in other countries (Bourne, 2006). The most important cause of its not-realization could be the lack of an unique and simple screening test: the best sensitivity-specificity balance, with a low cost-effectiveness ratio, seems to be achieved by combining various parameters and tools, such as IOP measurement, perimetry, CCT, and vertical CDR (Francis et al., 2011). Furthermore, screening for glaucoma has been demonstrated to be more cost-effective in targeted populations at high risk of glaucoma, such as older adults, patients with familiar history of glaucoma, and African American and Hispanics, rather than in general population (Friedman et al., 2004).

**FIGURE 8:**
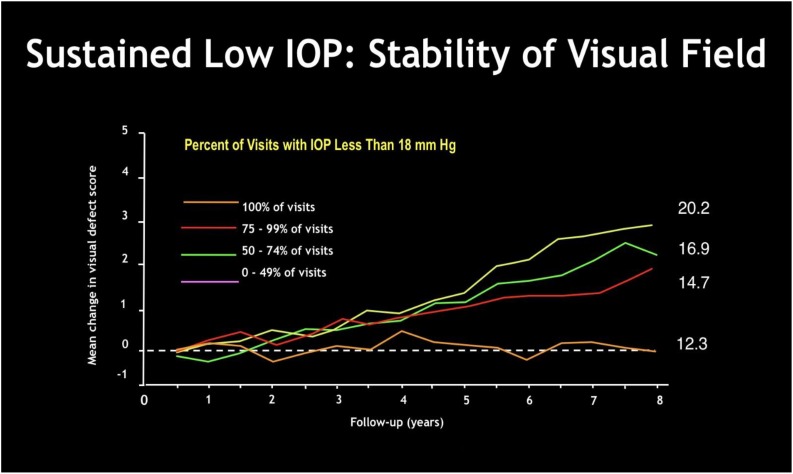
The Advanced Glaucoma Intervention Study (AGIS Investigators, 2000): sustained low IOP gives visual field stabilization over time. The greater is the percentage of examinations with IOP less than 18 mmHg, the smaller is the mean change in visual field defect score over months.

**FIGURE 9:**
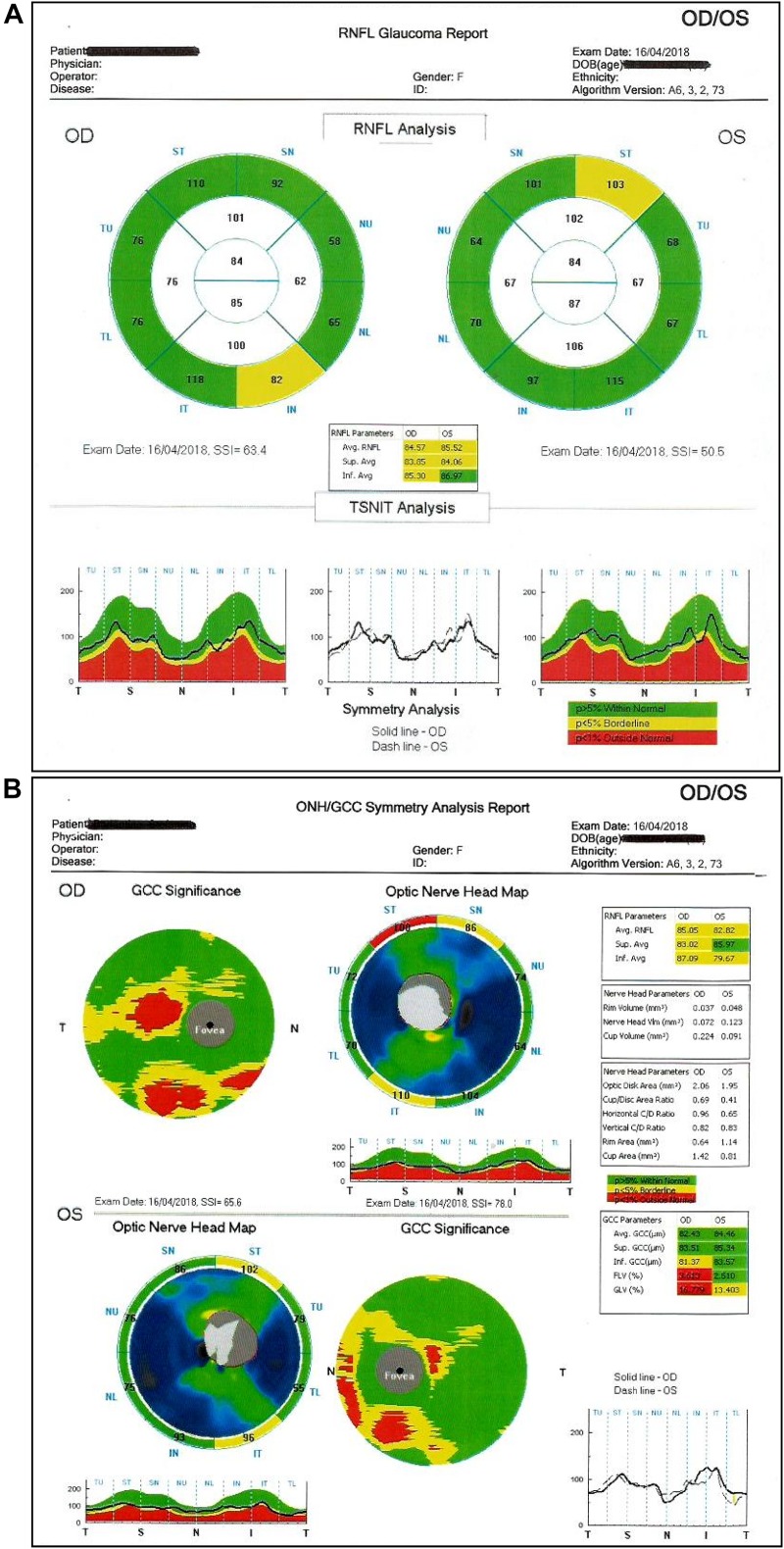
**(A,B)** Optical Coherence Tomography (OCT): it evaluates the status of the optic papilla, nerve fibers (RNFL) and ganglion cell layer.

Glaucoma is a chronic disease whose care pathway requires quick and easy access to clinical and instrumental controls, including IOP measurement, that is essential in order to prevent glaucoma. Despite the limitations described above, there is no doubt about the role of IOP in the development and progression of the disease. It is universally recognized that reducing IOP, regardless of other risk factors, decreases the risk of disease progression (Table 1). In doubtful cases, there are provocative tests, such as the ibopamine test, the water-drinking test and contrast sensitivity examination. In addition, the IOP maintenance below 18 mmHg ensures the stability of the visual field at 8 years of follow up (Figure 8). However, IOP is always related to the response and stress of ocular anatomical structures of the subject under examination. The other essential tests to perform are a visual field test, a diurnal tonometric curve and an overall eye examination every 3–5 months, depending on the stage and evolution of the disease, with instrumental evaluation of the morphology of the optic nerve head through HRT, GDx and OCT execution (Lin et al., 2007; Chauhan et al., 2008; Leung, 2014) (Figures 9A,B).

**TABLE 1:** Summaries of studies (Kass et al., 2002; Leske et al., 2003; Miglior et al., 2007; Chauhan et al., 2008; Garway-Heath et al., 2015) demonstrating that lowering IOP reduces the risk of progression of glaucoma, with the associated Hazard ratio.

Lower IOP reduces risk of progression		

Trial	Reduction of risk	Hazard ratio
EMGT^2^	10–13% per mmHg	0.90 (0.86–0.94)
OHTS^3^	10% per mmHg	1.11 (1.04–1.17)
EGPS^4^	12% per mmHg	1.12 (1.03–1.23)
CGS^1^	19% per mmHg	1.19 (1.05–1.36)
UKGTS^5^	19% mmHg	0.44 (0.29–0.69)

*1. Chauhan et al. Ophthalmol. 2008. 2. Leske et al. Arch Ophthalmol. 2003. 3. Kass et al. Arch Ophtalmol. 2002. 4. Miglor et al. Ophthalmology 2007; 114:39. 5. Garway-Health et al. Lancet 2014: December 19th e-pub.*

Considering, however, that the functional and anatomical damages due to glaucoma are mostly irreversible and that first defects of the visual field can be only found with traditional perimetry when 40% of ganglion cell fibers are already damaged (in necrosis or apoptosis), early detection remains an important strategy to prevent vision loss (Sommer et al., 1991). All this has led to the development of advanced ophthalmoscopic morphological methods to identify as early as possible loss of visual nervous tissue, such as OCT for optic papilla and retinal nerve fibers (Grewal and Tanna, 2013; Kostanyan et al., 2015; Kuang et al., 2015) and OCT angiography (without injection of contrast medium).

Another important early detection instrument is electroretinography (ERG), that allows to objectively quantify the retinal function by detecting retinal electrical responses. In addition, electrical stimulation has been performed as a novel approach to decrease IOP in open-angle glaucoma (Gil-Carrasco et al., 2018). Preclinical studies on gerbil eyes demonstrated that early transcorneal electrical stimulation (TcES) has a positive effect on RGC survival after retinal injury related to acute ocular-hypertension, protecting these cells from damage through modulation of the inflammatory response activated by microglial cells (Fu et al., 2018). Transpalpebral electrical stimulation (TES) performed on human eyes (Gil-Carrasco et al., 2018) has been proven to significantly lower IOP in eyes with open-angle glaucoma. The aim of TES is to reproduce the role of tyrosine kinase inhibitors, stimulating the reactivation of Ca^2+^ channels in the TM cells and so promoting their relaxation in order to promote the outflow of aqueous humor to the Schlemm’s channel. According to our experience, the functional damage of the trabecular cells in glaucoma is inversely proportional to the electrical stimulation of effectiveness. Less trabecular function in more advanced glaucoma results in less efficacy of the procedure and greater necessity to replicate it. Therefore, it is our opinion that electrical stimulation could be more useful in early stages of the disease. This concept further underlines the importance of early detection. Additional studies are needed to evaluate the maintenance of the IOP lowering effect over time after the treatment. Considering the above, molecular targets play an increasingly important role in the field of early detection and treatment of glaucoma (Chitranshi et al., 2018). It has been proven that RGC death is due to neurotrophic factor deprivation, gene dysregulation, hypoxia and excitotoxicity, as well as gene dysregulation and activation of apoptotic pathways. Therefore, studies focused on the possibility of enhancing signaling of BDNF-TrkB (brain-derived neurotrophic factor-tyrosin protein kinase) and on the chance to pharmacologically modulate TrKB. Endogenous phosphatase Shp2 has also been studied, considering its role in regulating TrKB. Recent findings showed that stress induced protein aggregation could cause the formation of unfolded proteins in the ER (endoplasmic reticulum) and apoptosis. In order to modulate the equilibrium between the apoptotic and the survival pathways, also proapoptotic Bcl2 and antiapoptotic Bax molecules are under investigation. Finally, despite its multifactorial etiology, a genetic association of POAG has been recognized and is still under observation. NTF4 and BDNF mutations are associated with the disease, as well as MYOC, OPTN, WDR36, and TMCO1 genes mutations. Further studies are needed to demonstrate a statistic significance of these mutations in the development of glaucoma, however, it is believed that they have a potential role in the early detection and therapy strategies of the disease. These evaluations, which could be employed as potential performance in the clinical-biological research of advanced glaucoma, could only be carried out in highly specialized glaucoma centers. Considering their costs, their application is recommended in cases of difficult diagnosis. These particular tests should be used to achieve a preventive diagnosis as soon as possible and avoid glaucomatous damage which, when established, could not be restored, but its progression could be arrested. Despite these challenges, other population based-studies have to be carried out to develop screening programs and the establishment of a concrete prevention plan is urgently needed.

## “Hub and Spoke” Management of Glaucoma

The term “Hub and Spoke” is borrowed from the vocabulary used in aviation. “Hub and spoke” is a model to develop the airline network, consisting of a hub where most flights are concentrated. The term was created by analogy with the bicycle wheel (*hub* = center, *spoke* = radius).

**FIGURE 10:**
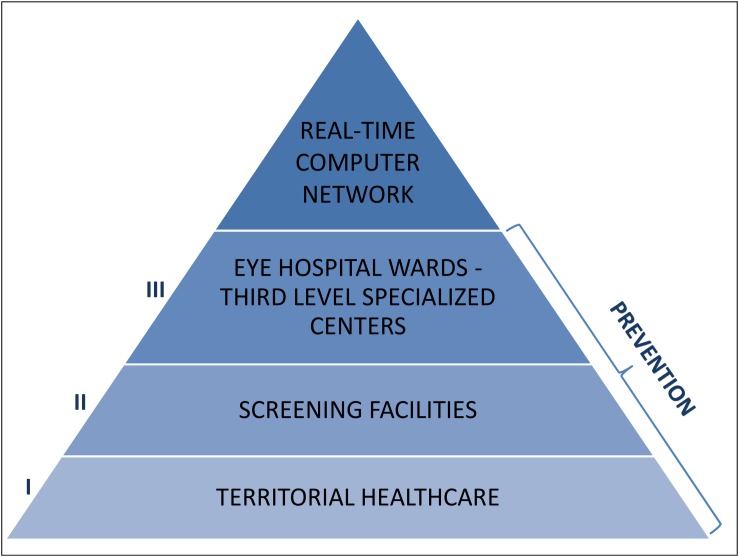
Glaucoma prevention, where and how: the primary prevention should be mainly a competence of the territorial healthcare, the secondary prevention should be carried out by screening facilities and the third prevention should be operated by the Eye hospital wards and the third level specialized centers. The essential link that unites and connects all these identities shall be the creation of a real-time computer network.

Thus, in the field of glaucoma prevention, it is necessary to set up a communication network, enabling informed people to be effortlessly conveyed into screening programs or health facilities (if disease is already diagnosed), taking into account the status of the glaucomatous disease and without losing any particular of the patient damage in the coded path (Figure 10).

**FIGURE 11:**
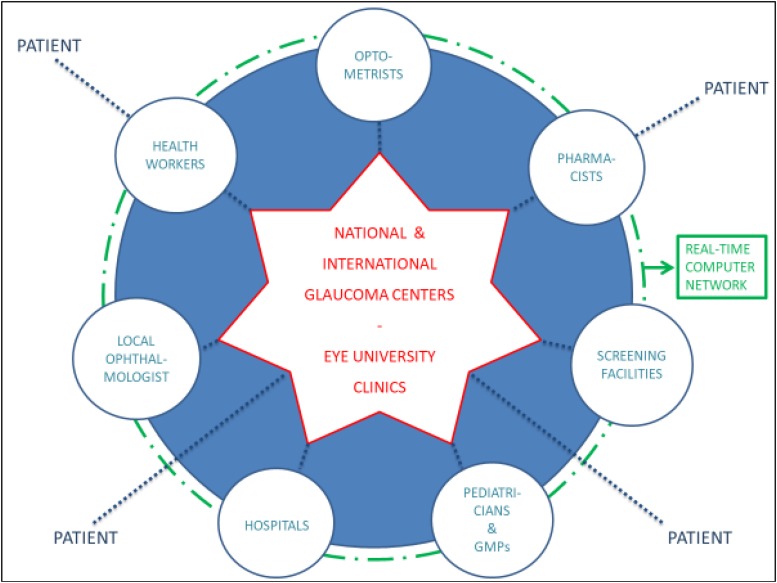
The “hub and spoke” model of glaucoma management: the hubs correspond to optometrists, pharmacists, pediatricians, general medical practitioners (GMPs), the local ophthalmologist, health workers, screening facilities and hospitals, while the spoke is represented by the national and international glaucoma centers and the Eye University Clinics.

According to the “Hub and Spoke” theory (Elrod and Fortenberry, 2017), patients must be directed to specialized establishments (hubs), toward well-organized peripheral health structures (spokes): the national and international anti-glaucoma centers correspond to the hubs, while screening facilities, territorial healthcare and hospitals match the spokes. The communication network shall be also realized at various levels between health professions (opticians, optometrists, pharmacists, and family doctors) and non-health workers (teachers, employers, communities and recreational centers, non-healthcare personnel working in INAIL or INPS) (Figure 11). The performance of certain diagnostic procedures may be delegated to properly trained personnel, then the interpretation of the results and the associated medical and surgical decisions require the experience of the ophthalmologist. Most therapeutic procedures can be carried out ambulatory, although in some instances hospitalization may be required and it is important to educate patients in the management of their condition. The ultimate objective is to facilitate glaucoma prevention through information and awareness campaigns, such as leaflets, posters, meetings, footages, TV spots, healthcare information stands, the organization of The World Glaucoma Week (the last one was the one of March 10–16, 2019) and also screening consulting rooms in healthcare environment or not: for example, mobile check points equipped in squares on selected screening days, such as The Sight and Glaucoma World Days and festivities. Patients should be encouraged to communicate to their ophthalmologists or family doctors not only variations of their clinical conditions, but also their emotional changes (and eventual fear of blindness) during topical hypotensive therapy. Patients with substantial visual impairment or blindness can be referred for appropriate vision rehabilitation and social services and those considering keratorefractive surgery should be informed about the possibility of reduced contrast sensitivity and decrease accuracy of IOP measurements after laser vision correction (Shin et al., 2015).

Furthermore, it is necessary to develop and use more and more advanced diagnostic methods, such as the various technologies of eye hemodynamics and Echocolourdoppler, the Heidelberg retinal flowmeter, the short wavelength automated perimetry (SWAP or blue-on-yellow perimetry), the high-pass resolution perimetry, the frequency doubling technology (FDT) perimetry, the pattern ERG, the chromatic visual evoked potential (CVEP), the detection of apoptosing retinal cells (DARC, that could be used as a biomarkers in the follow up of glaucoma), the telemetric contact lenses (recording IOP fluctuations over 24 h) and the genetic tests (currently 65 genes in multifactorial polygenic model are known) (Bowling and Kanski, 2016). These diagnostic tools shall be applied taking account of the environmental risk factors to define an accurate individual risk profile.

In the end, the only hub-and-spoke management can lead to focus treatment of this serious social disease with an adequate and efficient sanitary network. Indeed, glaucoma is increasingly insidious and widespread and it must be approached with a neuro-restorative attitude, becoming more and more clinicobiological, especially in relation to prevention and plastic condition of peripheral and central nervous system (Nuzzi and Tridico, 2017; Nuzzi et al., 2018).

## Discussion

The purpose of early diagnosis and treatment of glaucoma is to preserve the residual sight from any further deterioration, since it is not possible to regain what has been lost.

Therapeutic–preventive struggle against this devious disease should not be underestimated and implemented extensively for the following directives:

– anti-glaucomatous propaganda, in particular by the general medical practitioner (GMP);
– sending of at-risk subjects and suspected glaucoma patients to specialized antiglaucomatous centers, also by GMPs;
– development of telemedicine and teleglaucoma, especially in places where patients have poor access to diagnosis and treatment, as well as prevention;
– evidence of coexistence of glaucoma with other systemic diseases, particularly for the progressive increase in average life;
– medical approach focused on drug-drug interactions: for example, antihypertensive drugs reduce IOP, while corticosteroids, antidepressant drugs and tranquilizers increase it; carbonic anhydrase inhibitors stimulate ventilation and therefore accentuate dyskinesis in patients with various bronchopulmonary diseases, while the administration of beta-blocker eye drops in patients with heart failure results in asthenia with dyspnea and bradycardia, that may lead to erroneous cardiovascular conclusions;
– it is necessary to establish an ever closer collaboration between the ophthalmologist, the general practitioner and the other specialists for the good and interest of patients, also through the creation of an updated computer network (database) that can be consulted in real-time;
– the GMP must know that all glaucoma patients need long-term therapies that are often associated with systemic therapies, as well as he should report if patient lack of compliance is present;
– the family doctor has the crucial task to identify glaucoma risk factors, including familiarity, subjects age, diabetes, high blood pressure and hyperlipidemia;
– from an interdisciplinary perspective, all doctors shall refer all patients at risk to make an ophthalmologic screening, especially focusing on tonometry and perimetry; this is important because the cost-benefit ratio increases when such screening is implemented in patients aged between 40 and 60, as already describe above; and
– it is required to know and explain how cataract surgery, when required, lowers IOP significantly by 3–4 mmHg and clearly improves the vision, as well as the optic nerve head monitoring and the obtainable perimetric data.

Moreover, in health education, anyone should obey the following precautions:

– do not wear tight collars or clothes (that are too tight) around the neck;
– do not keep the head down for a long time;
– no drinking copious amounts of liquids in a short time;
– do not drink coffee and smoke more than five cigarettes a day or halve the use of electronic cigarette;
– avoid constipation; and
– avoid repeated psychic emotions and excessive physical fatigue, as well as the consequent excessive use of antidepressants or tranquilizers.

In conclusion, it is therefore essential to create an high-performance pyramidal “Hub and Spoke” system, that will have as central figures on the territory the optometrist, the GMP and the district nurse, the ophthalmologist and the territorial outpatient optometrist, while the hospital landmarks will be the eye hospital wards and the third level specialized centers. This organization, with the constant support of telemedicine and real-time computer network, will inevitably lead to an ever greater stabilization of this chronic disease in the early stages (detecting increasingly favorable epidemiological and statistical evidence). Furthermore, it will have undoubted social implications thanks to the preservation of the eyesight, that after life is the most important function to modern society.

## Author Contributions

All the authors gave an equal contribution in a balanced way to the realization of the article, in particular for the abstract and discussion, as well as for the subchapters: Introduction, Glaucoma Diagnosis, Eye Diseases Prevention, Glaucoma Screening, “Hub and Spoke” Management of Glaucoma.

## Conflict of Interest

The authors declare that the research was conducted in the absence of any commercial or financial relationships that could be construed as a potential conflict of interest.
